# Chromophore Planarity, –BH Bridge Effect, and Two-Photon Activity: Bi- and Ter-Phenyl Derivatives as a Case Study

**DOI:** 10.1021/acs.jpca.3c04288

**Published:** 2023-09-18

**Authors:** Swati Singh Rajput, Robert Zaleśny, Md Mehboob Alam

**Affiliations:** † Department of Chemistry, Indian Institute of Technology Bhilai, GEC Campus, Sejbahar, Raipur, Chhattisgarh 492015, India; ‡ Faculty of Chemistry, Wrocław University of Science and Technology, Wyb. Wyspiańskiego 27, PL-50370 Wrocław, Poland

## Abstract

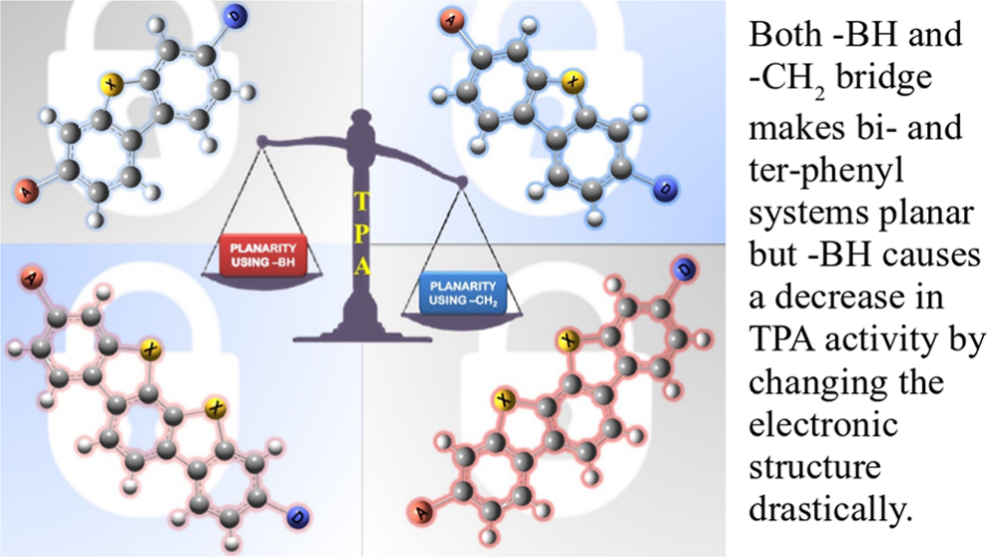

In this work, we have employed electronic structure theories to explore the effect of the planarity of the chromophore on the two-photon absorption properties of bi- and ter-phenyl systems. To that end, we have considered 11 bi- and 7 ter-phenyl-based chromophores presenting a donor−π–acceptor architecture. In some cases, the planarity has been enforced by bridging the rings at ortho-positions by −CH_2_ and/or –BH, –O, –S, and –NH moieties. The results presented herein demonstrate that in bi- and ter-phenyl systems, the planarity achieved *via* a −CH_2_ bridge increases the 2PA activity. However, the introduction of a bridge with the –BH moiety perturbs the electronic structure to a large extent, thus diminishing the two-photon transition strength to the lowest electronic excited state. As far as two-photon absorption activity is concerned, this work hints toward avoiding –BH bridge(s) to enforce planarity in bi- and ter-phenyl systems; however, one may use −CH_2_ bridge(s) to achieve the enhancement of the property in question. All of these conclusions have been supported by in-depth analyses based on generalized few-state models.

## Introduction

Two-photon absorption (2PA) was predicted in the 1930s by Maria Göppert-Mayer, and nowadays, one witnesses its numerous applications including biological imaging,^1,2^ photodynamic therapy,^3,4^ three-dimensional (3D) microfabrication,^5^ and 3D optical data storage.^6,7^ With these applications in mind, there is ongoing research regarding the development of two-photon^8−10^ and three-photon absorbers.^11,12^ Based on structure–property relationships, it has been observed that significant 2PA cross sections can be achieved in systems that exhibit some of these characteristics such as noncentrosymmetry,^13,14^ planarity,^15−17^ donor–acceptor interactions,^18−23^ or extended π-conjugation.^24−27^ Studies have revealed that planarity in the π-conjugated system leads to better π-orbital overlap, which is beneficial for nonlinear absorption cross sections corresponding to transitions to low-lying states as it facilitates charge transfer upon electronic excitation. Fang et al. studied the 2PA activity of *p*-triphenyl-based dendrimer systems, and it was observed that the planar form exhibited enhanced 2PA.^28^ An interesting study on the relation between 2PA and π-conjugation pathway was carried out by Ahn et al.,^29^ who considered porphyrin-based moieties in various orientations. These authors concluded that the planar systems deliver the highest 2PA activity.

In order to shed more light on the relation between system planarity and (non)linear absorption, the present study focuses on two families of organic compounds, namely, biphenyl (BP) and *p*-ter-phenyl (TP) systems. BP and its derivatives are naturally present in coal tar, natural gas, and crude oils and can be isolated through distillation.^30^ In contrast, TP and its derivatives are naturally present and extracted from some species of fungi.^31^ These systems have a more comprehensive range of applications; for example, BPs have been used as heat transfer fluids^32^ and food preservatives^33^ and in the production of polychlorinated biphenyls.^34^ At the same time, TPs have found their applications as components of organic light emitting diodes,^35^ therapeutics,^36−38^ and catalysts.^39^

BPs and TPs were also extensively studied to understand their environment-dependent structure. Bastiansen et al.^40^ performed the electron diffraction study of BPs demonstrating that in the gas phase, these are nonplanar and have a dihedral angle of 45°. The nonplanar *ortho*-substituted BP derivatives are optically active.^41^ X-ray study of the crystal of TPs under room temperature revealed that they are coplanar;^42^ however, TPs show nonplanarity in the solvent phase.^43^ Numerous studies were carried out on BP and TP derivatives that exhibit nonlinear optical properties (NLO),^44^ including multiphoton absorption. Specht et al. engineered BP-based donor–acceptor systems that were characterized by large values of the 2PA cross section.^45^ In 1974, Drucker et al. experimentally demonstrated the 2PA in TP systems using polarization technique.^46^ Morikawa et al. studied the 2PA coefficient of TP crystal by using a charge carrier generation mechanism.^47^

Studying the nonlinear optical properties of conformationally (un)locked molecular systems, e.g., with locking achieved by hindering rotation along the single bond, might contribute to the development of structure–property relationships for designing materials with improved nonlinear optical characteristics. In fact, some studies focused on the effect of intramolecular charge transfer and its relation to first hyperpolarizability in conformationally locked systems. Van Walree et al. studied the electronic second-order NLO property (first hyperpolarizability) of BPs with enforced planarity through conformational locking.^48^ The study in question revealed that these systems have a small/negligible increase in hyperpolarizability as compared to that of their unlocked counterpart.^48^ Hyunhee et al. studied the relationship between planarity and intramolecular charge transfer. To that end, these authors synthesized TP rings in a planar and distorted form and observed that the intramolecular charge transfer was higher for a system that is planar.^49^ Ruud et al.^50^ have found that the 2PA of aryl-substituted BODIPY is maximized when the two ring systems are at a dihedral angle of ∼50°. There are very few works where the effect of planarity of rings on 2PA activity has been thoroughly analyzed, and this motivates the present study. In more detail, the goal of the present work is to employ electronic structure calculations to make a link between the 2PA activity of BP and TP systems and their conformation and structural parameters. To that end, we will employ electronic structure calculations to study a number of donor–acceptor *para*-substituted BP and TP molecules, where the two rings either show rotational freedom or are clamped to restrict the rotation. The emphasis will be on the *S*_0_ → *S*_1_ dipole-allowed transition, as usually, multiphoton excitations to higher excited states do not present application-related potential.

## Computational Details

**Figure 1:**
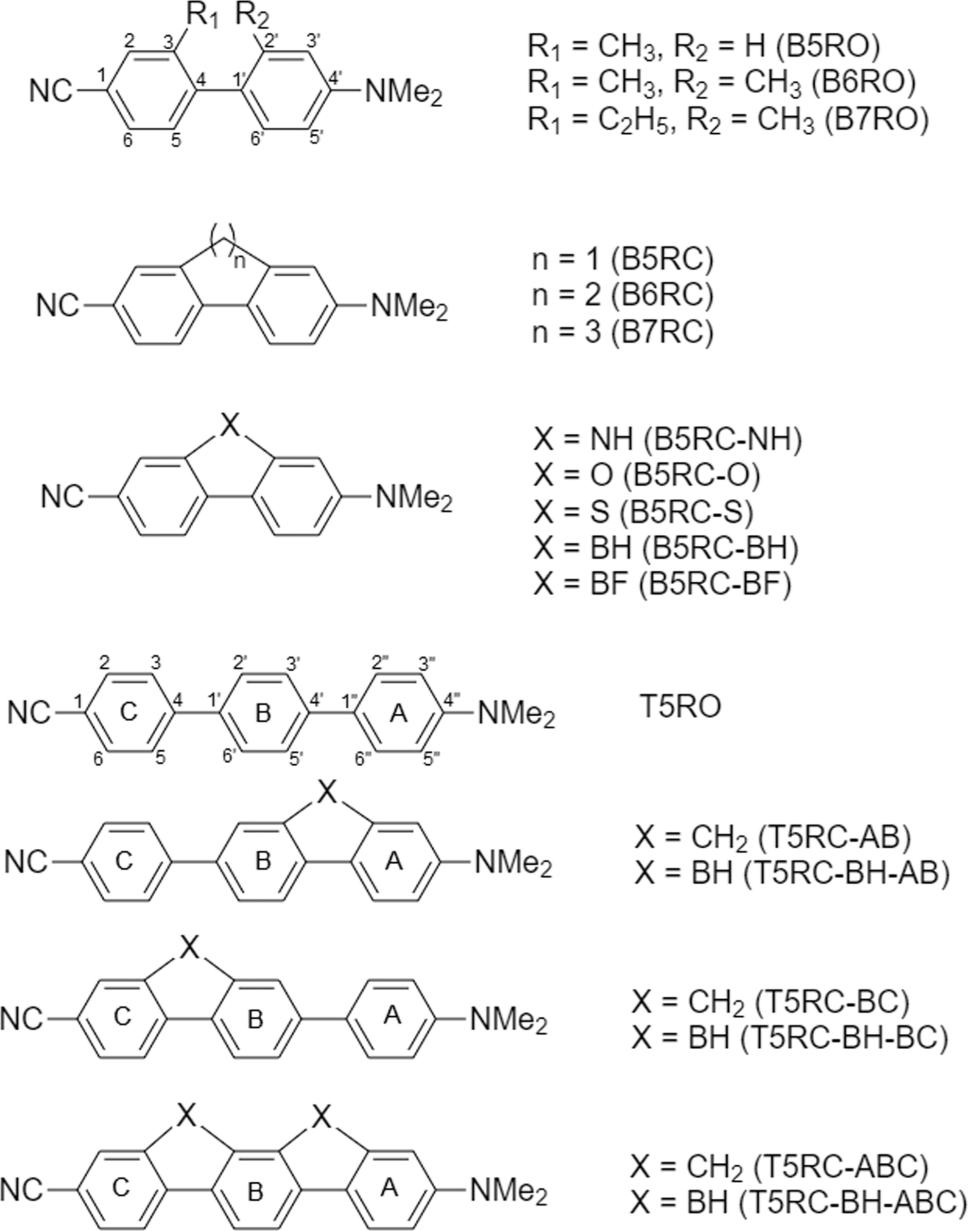
Molecules considered in this work.

The systems considered in this work, together with the atom and ring labels, are shown in Figure 1. We will consider a total of 11 BP-based and 7 TP-based molecules. The ground-state geometry of all molecules was optimized in the gas phase at B3LYP/6-311+G(d,p) level of theory^51^ using the Gaussian 16 program.^52^ The positive definite nature of the corresponding Hessian confirmed that the optimized structures truly belong to the minima on the potential energy surface. Subsequently, the one- and two-photon absorption strength corresponding to the transitions to the *S*_1_ state in all of the structures were calculated at the RI-CC2/cc-pVDZ level of theory^53−57^ as implemented in the Turbomole 7.3 program.^58,59^ In order to link the 2PA transition strengths with electronic structure, the generalized few-state models were employed, and the relevant properties such as excited to excited-state transition moments and excited-state dipole moments were calculated for all of the systems at the same level of theory. Moreover, the electronic density difference plots and charge-transfer metric^60,61^ (*d*_CT_) were determined at MN15/6-311+G(d,p) level of theory^62^ using the Gaussian 16 program. It is important to mention here that in a recent work by Grabarz et al., it has been reported that MN15 functional with a substantial amount of exact exchange delivers the accurate ground- and excited-state electronic density distribution as compared to the coupled-cluster CC2 model.^63^ Based on these findings, we chose MN15 for electronic density analysis in this work.

## Results and Discussion

### Structural Studies

All of the molecules considered in this work were studied assuming a *C*_1_ symmetry point group. We will start this section with a discussion of the major geometrical features of these compounds. The dihedral angles between adjacent rings for all studied systems are given in Table 1. The variation of dihedral angles is explicable based on the freedom of rotation of the rings involved. For instance, in B7RC, the two rings connected *via* –(CH_2_)_3_– linkage have more conformational freedom than that in B5RC (or B6RC) and hence the relevant dihedral angle between the two phenyl rings is larger in the former than in the latter two compounds. Similarly, in B5RC-X (X = –NH, –O, –S, –BH, –BF), the two rings are fixed at their positions by a single bridging group (the –X group) as in B5RC and hence the rings are bound to be coplanar. At the same time, the TP system in its open form, *i.e.*, T5RO, where all of the rings show rotational freedom along the C–C bond, possesses a larger dihedral angle as compared to the systems where any two rings are blocked by attaching an –X group. When all of the rings are attached by bridging groups, the corresponding dihedral angle tends to be zero, thus making the system planar.

**Table 1:** C3–C4–C1′–C2′ and/or C3′–C4′–C1″–C2″ Dihedral Angles, *S*_0_ → *S*_1_ Excitation Energy in [eV] (Wavelength in [nm]), and δ_2PA_^Resp^ in [au] of All of the Systems Considered in This Work^a^

system	dihedral angle (deg)	excitation energy [eV] (wavelength in [nm])	δ_2PA_^resp^(×10^3^ [au])
B5RO	–54.4	4.36 (285)	14.5
B6RO	–89.4	4.52 (274)	0.01
B7RO	–108.8	4.48 (277)	5.0
B5RC	0.0	3.99 (310)	16.5
B6RC	–19.7	3.95 (314)	20.2
B7RC	–46.4	4.16 (298)	16.7
B5RC-NH	0.0	3.96 (313)	11.1
B5RC-O	0.0	3.97 (313)	13.3
B5RC-S	0.0	3.95 (314)	15.9
B5RC-BH	0.0	2.28 (544)	4.1
B5RC-BF	0.0	2.72 (456)	4.3
T5RO	37.79, 36.36	4.07 (305)	45.9
T5RC-AB	37.46, 0.22	3.84 (323)	47.8
T5RC-BC	–0.23, −36.49	3.93 (316)	46.9
T5RC-ABC	–0.005, −0.01	3.72 (334)	49.8
T5RC-BH-AB	–37.3, −0.3	2.28 (545)	4.1
T5RC-BH-BC	–0.2, −36.0	2.64 (470)	14.8
T5RC-BH-ABC	0.0, −0.1	2.00 (621)	1.9

a Ground-state geometry of all of the systems is optimized at B3LYP/6-311+G(d,p) and spectroscopic properties are calculated at the RI-CC2/cc-pVDZ level of theory.

### One-Photon Absorption

In BP systems, we noticed that as the C atoms are added simultaneously in B5RO → B7RO, respectively, there is no significant change in the position of the one-photon absorption (1PA) peak corresponding to the *S*_0_ → *S*_1_ transition. In comparison to open systems, the closed ones absorb at slightly higher wavelengths. However, there is no significant change in the 1PA wavelength when the dihedral angle between the two rings is increased on passing from B5RC to B7RC. Similarly, all of the B5RC-X systems with –X as –O, –NH, and –S have the same 1PA wavelength. Interestingly, B5RC-BH and B5RC-BF, although have a zero dihedral angle, absorb at longer wavelengths (∼550 and ∼460 nm, respectively). This change is not related to the dihedral angle between the rings, but it could be correlated with the presence of an empty p-orbital on the bridging B atom. This feature causes a significant alteration of the electronic structure of the system reflected in their 1PA wavelengths. Similarly, in TP systems, if the rings are closed with the –BH unit, a substantial increase in 1PA wavelength is observed as compared to when the rings are closed by the −CH2 unit. The results demonstrate that the 1PA wavelength is also sensitive to the position of the bridge between rings A (containing a donor group) and B or between rings B and C (containing an acceptor group). Usually, the system, with the donor-group-containing ring coplanar with the middle ring, absorbs at a higher wavelength than the system where the acceptor-group-containing ring is made planar to the middle ring. The systems with all of the rings planar to each other absorb at the longest wavelength in their group.

To get further insight into the nature of the *S*_0_ → *S*_1_ transition in all these systems, we analyzed the involved orbitals. The orbital pictures are shown in the Supporting Information. We noticed that for all of the systems, except B6RO, the *S*_0_ → *S*_1_ excitation is dominated by the highest occupied molecular orbital (HOMO) → lowest unoccupied molecular orbital (LUMO) transition. In B6RO, the main contributions come from HOMO → LUMO + 2 (42%), HOMO → LUMO + 1 (24%), and HOMO → LUMO (21%) transitions. It should be noticed that in the case of B6RO, the *S*_0_ → *S*_2_ transition is dominated by the HOMO → LUMO transition. This indicates that the nature of the *S*_2_ state in B6RO is the same as that of the *S*_1_ state in other systems. This needs to be noted for our further observations and analysis. The orbital images depict that the spatial distribution of HOMO and LUMO are well separated when the two rings are oriented at a nonzero dihedral angle and the two are mixed in planar systems. The said mixing patterns are the same when the rings are closed either by the −CH_2_ unit or by a heteroatom such as X = –NH, –O, and –S. However, when X = –BH or –BF, the LUMO electron density is more localized on the bridging atom. This clearly reflects the change in electronic structure induced by the presence of the B atom. Note that in the case of the B5RC-S, the electronic density of the S atom has a large contribution to HOMO and that does not hold for the C, N, and O atoms in B5RC, B5RC-NH, or B5RC-O, respectively. One can conclude that except when the bridging group is –BH or –BF, the one-photon *S*_0_ → *S*_1_ transition does not depend significantly on the bridging atom.

**Figure 2:**
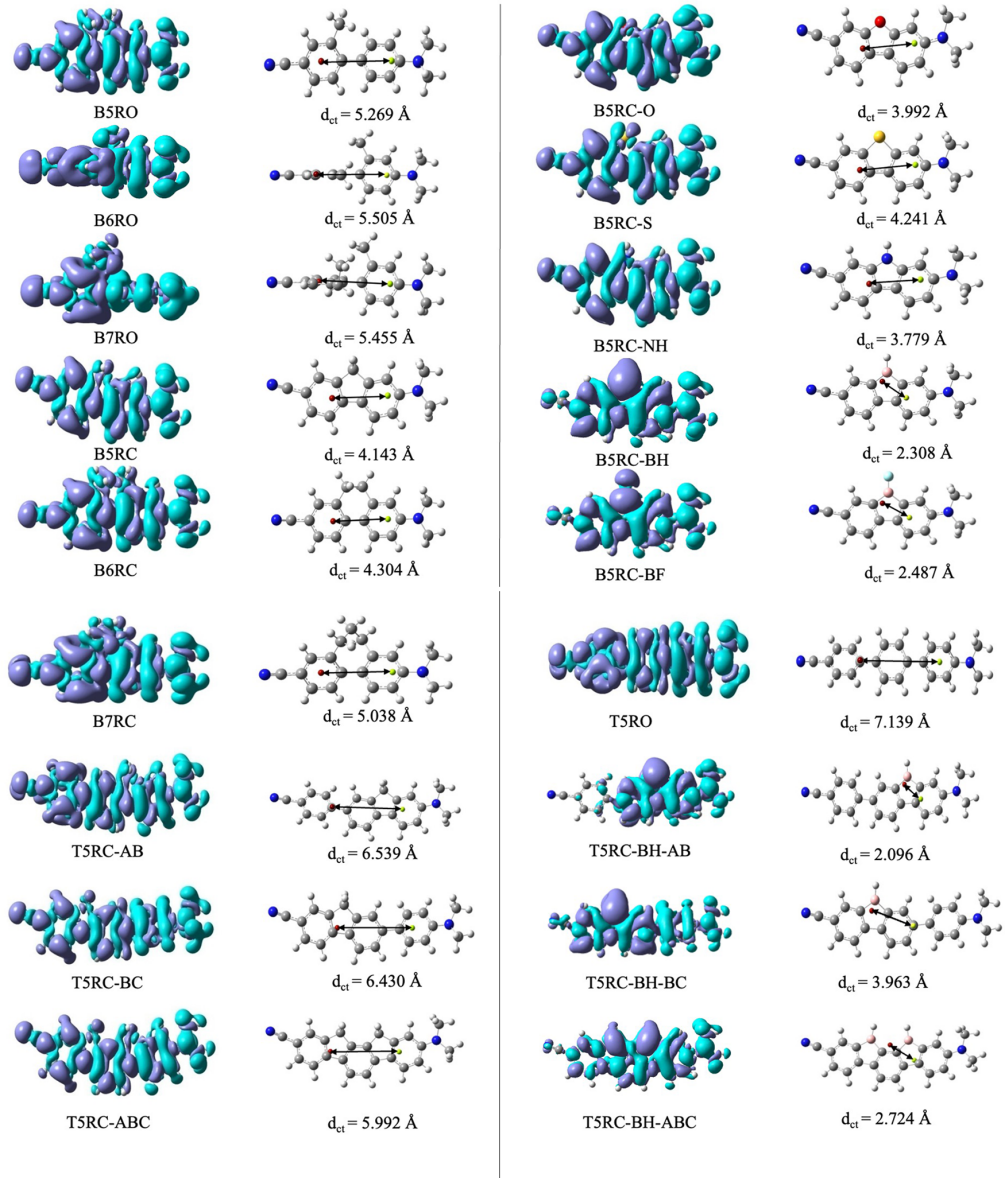
Graphical representation of density difference between *S*_1_ and *S*_0_ states: turquoise implies electron-access zone and blue represents electron-deficient zone; *d*_CT_ (numerical values given in Å unit) and *R*_+_/*R*_–_ (represented by yellow and red dots, respectively). All these are computed at MN15/6-311+G(d,p) level. For the density difference plot isocontour value of 0.0004 au is taken.

Let us now turn to the analysis of the character of electronic excitations. In order to obtain the quantitative characteristics of the extent of charge transfer in these systems, we calculated the distance (*d*_CT_) between the barycenter of positive and negative charge densities,^60,61^ given as
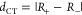
1where *R*_+_ and *R*_–_ represent the location of the barycenter of positive and negative charges, respectively. Along with *d*_CT_, we calculated the difference in the electron density between *S*_1_ and *S*_0_ states in each system. The density difference plots, barycenters, and *d*_CT_ for all of the systems are presented in Figure 2. The turquoise zone in Figure 2 represents the electron excess space, whereas the blue zone represents the electron-deficient space. The red and yellow dots in Figure 2 represent *R*_+_ and *R*_–_, respectively. We noticed that *R*_+_ and *R*_–_ are largely affected by the structure (open or closed form) of the molecule, the nature of the bridging atom, and the position of the bridge (as in TP systems). In general, open systems are found to have significantly larger *d*_CT_ values than their closed siblings. The order of *d*_CT_ in closed BP systems is B7RC (5.038 Å) > B6RC (4.304 Å) > B5RC (4.143 Å), which is consistent with the distance between the donor and acceptor groups in the molecules. However, in open BP systems, the order is B6RO (5.505 Å) > B7RO (5.455 Å) > B5RO (5.269 Å). In open systems, the planarity is lost, as evident from the values of dihedral angles (see Table 1). In B6RO, the two rings are now almost perpendicular to each other, which hampers the charger transfer drastically, and the C1′–C4 bond loses its double-bond character. This increases the distance between two terminals of the molecule, and hence B6RO has the largest *d*_CT_ value. When the rings in BP systems are closed using heteroatoms, the value of *d*_CT_ usually decreases (compared to B5RC) except B5RC-S (*d*_CT_ = 2.241 Å), where it is increased slightly. This is related to the electronegativity of the bridging atom/group. A more electronegative atom pulls the *R*_–_ toward itself, which decreases the distance between *R*_+_ and *R*_–_. Similarly, if the bridging group is –BH or –BF, due to the presence of an empty orbital on the B atom, *R*_+_ is shifted toward it, which decreases *d*_CT_. In fact, the effect of empty orbital of B atom on *d*_CT_ seems to prevail over other effects. We have not observed any strong dependency of *d*_CT_ on the planarity of the rings. Finally, let us conclude that similar results are obtained for TP systems.

### Two-Photon Absorption

The two-photon transition probability (δ_2PA_^Resp^) of all of the systems calculated using response theory at the RI-CC2/cc-pVDZ level is also given in Table 1. We made the following observations from these data.

1. The value of δ_2PA_^Resp^ for B6RO is much smaller than that of any other open or closed BP systems. Considering the excited-state character (see the previous section), we calculated δ_2PA_^Resp^ for the *S*_2_ state of B6RO; likewise, the corresponding 2P transition strength was found to be very small.
2. The closed BP systems (rings closed by −CH_2_ unit), where the dihedral angle between the two rings is zero or very small, have larger δ_2PA_^Resp^ than their open counterparts. The order of δ_2PA_^Resp^ in open and closed BP systems is opposite to each other, viz., B5RO > B7RO > B6RO and B5RC < B7RC < B6RC.
3. When the two rings in BP systems are closed by a heteroatom (X = –NH, –O, –S, –BH, –BF), the value of δ_2PA_^Resp^ decreases, which is drastic in the case of X = –BH and –BF.
4. In TP systems, when the rings are closed by the −CH_2_ unit, the value of δ_2PA_^Resp^ does not change much; however, when the same is done with the –BH unit, it decreases significantly (often by an order of magnitude). This decrease depends upon the position of the bridge, i.e., whether the bridge is between the donor-containing ring and the middle ring or between the acceptor-containing ring and the middle ring. The 2PA strength value decreases drastically if the donor-containing ring is made planar with the middle ring or if all of the three rings are made planar to each other. This reflects that planarity basically decreases the 2PA transition probability, at least when the bridge is made using the –BH unit.

**Figure 3:**
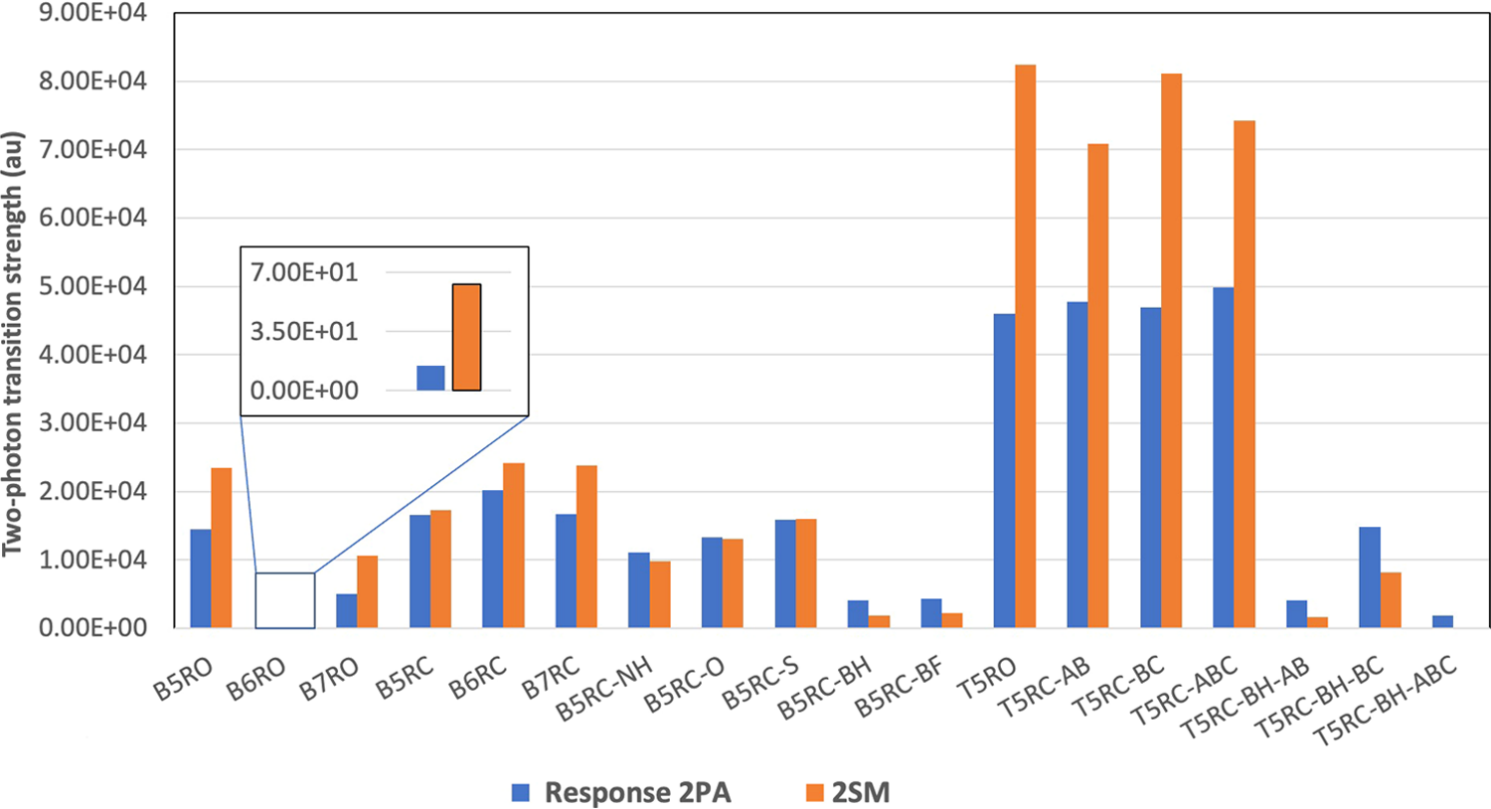
Two-photon transition strength, calculated at response theory (response 2PA), two-state model (2SM) with first excited state respectively as an intermediate state.

To explain these observations, we employed the generalized few-state model (GFSM) developed within the non-Hermitian framework.^64,65^ The corresponding expression of δ_2PA_^GFSM^ for the *S*_0_ → *S*_*f*_ transition is given as^64^
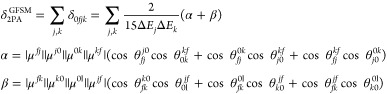
2where the summation indices j and k vary over all of the electronic states of the system, μ*^pq^* is the transition dipole moment vector for *S_p_* → *S_q_* transition,  with *E*_0*i*_ as *S*_0_ → *S*_*i*_ transition energy, and θ_*pq*_^*rs*^ is the angle between transition dipole moment vectors μ*^pq^* and μ*^rs^*. For our purpose, we employed a two-state model (2SM) including *S*_0_ and *S*_1_ states in all of the systems. The values of δ_2PA_^Resp^ and δ_2PA_^2SM^ for the *S*_0_ → *S*_1_ transition in all of the systems are plotted in Figure 3. The plot shows that for BP systems, 2SM essentially reproduces the trend of response theory results. For TP systems, in particular, for T5RO, T5RC-AB, T5RC-BC, and T5RC-ABC, 2SM overestimates the response theory results. However, 2SM reproduces the observation that there is no significant change in 2PA of these systems.

**Figure 4:**
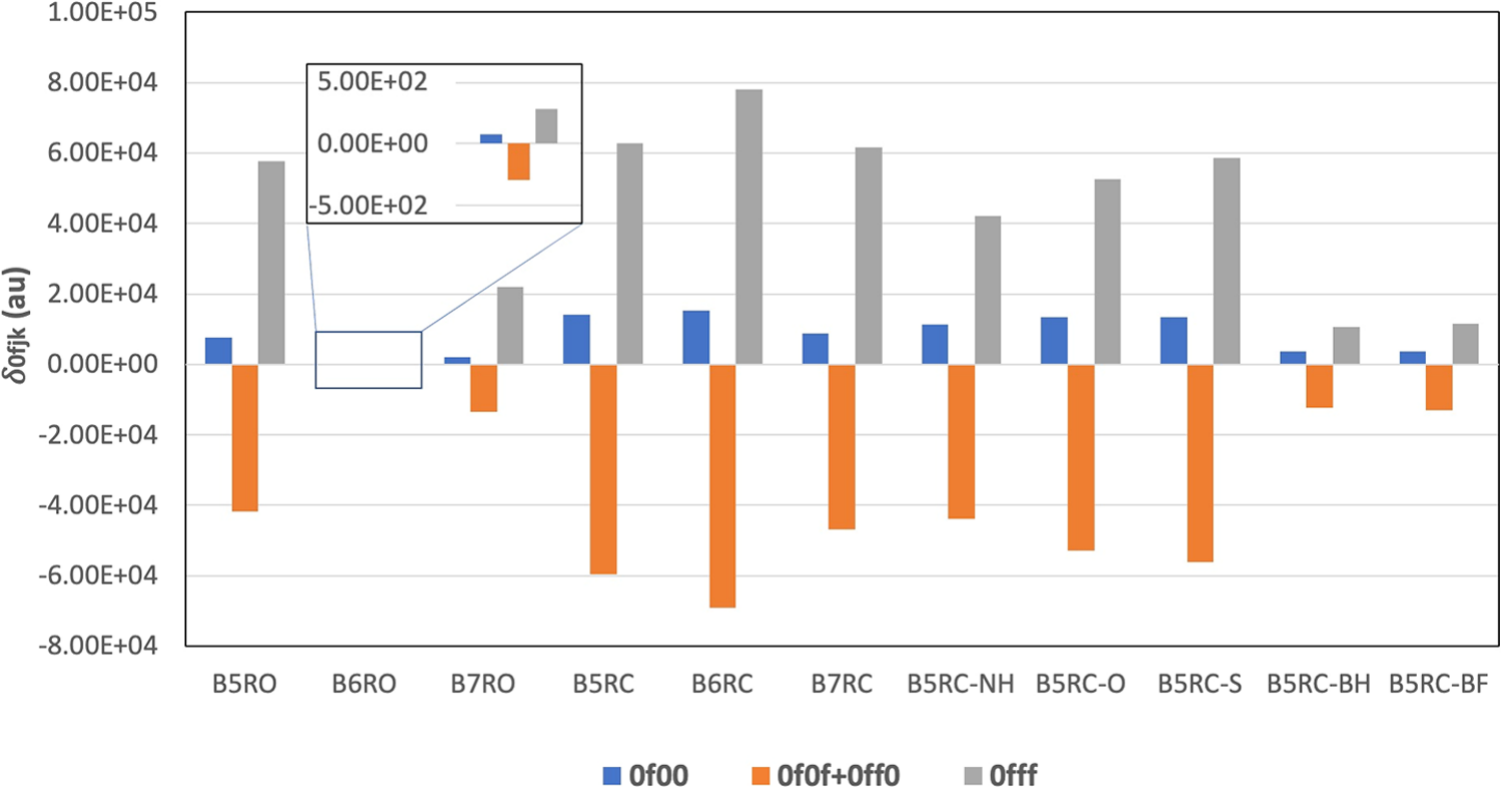
Values of different δ_0*fjk*_ terms involved in 2SM for all of the BP systems.

**Figure 5:**
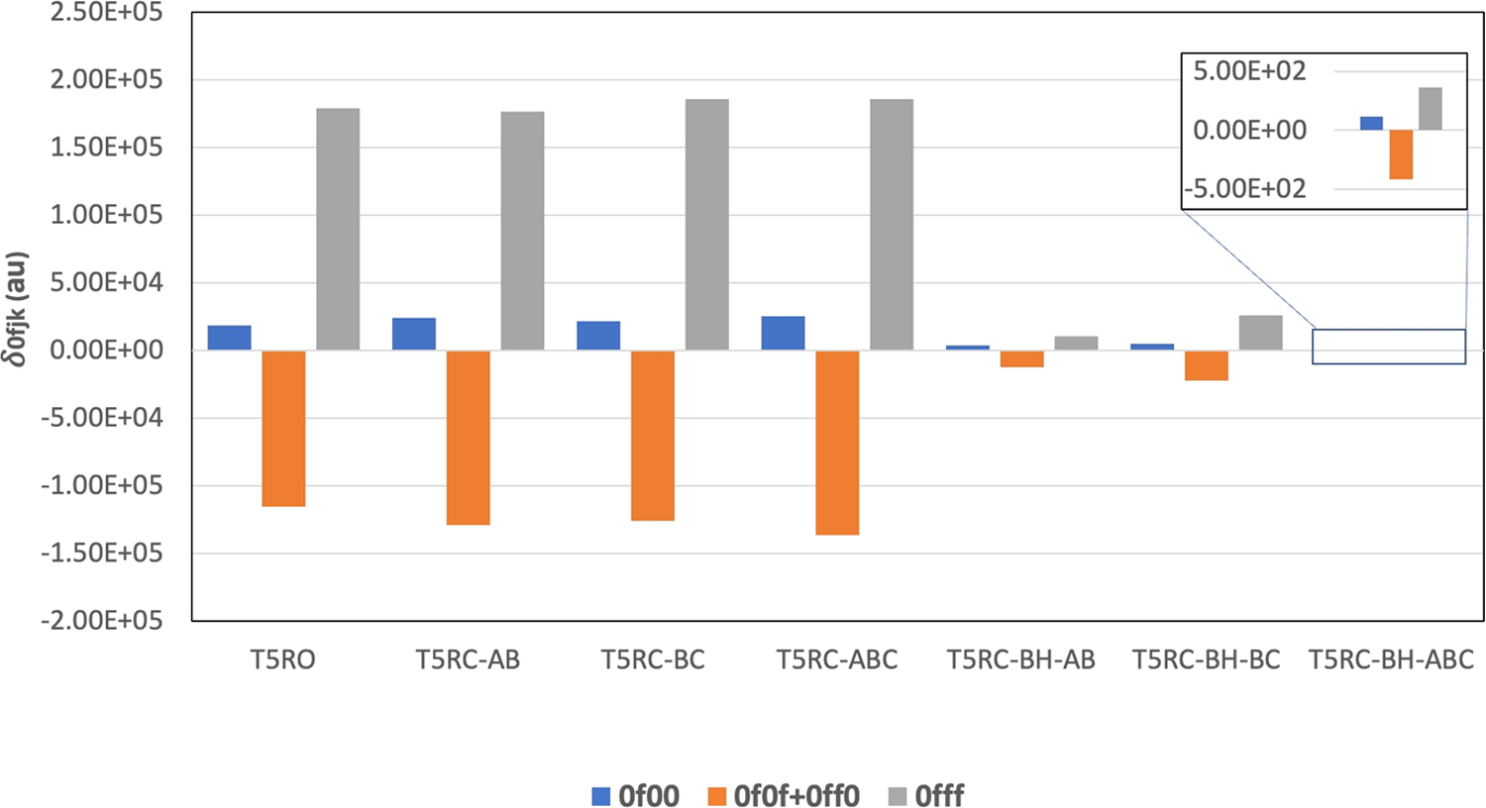
Values of different δ_0*fjk*_ terms involved in 2SM for T5RO, T5RC-X, and T5RC-BH-X systems.

Now, let us explain the observations we made above using 2SM results. The 2SM expression contains the following four terms δ_0100_, δ_0111_, δ_0110_, and δ_0101_. The first two terms are always constructive (positive), whereas the last two are always destructive (negative) in nature. Furthermore, the last two terms are identical to each other. For all of the BP and TP systems, the values of these four terms are plotted in Figures 4 and 5, respectively. The plot shows that in all of the systems, the positively contributing terms δ_0111_ and δ_0100_ are largely compensated by the negatively contributing δ_0101_ + δ_0110_. Among these, δ_0100_ has a very small value for all of the systems. B6RO has the smallest values for all of the four terms. Additionally, the negative contribution of δ_0101_ + δ_0110_ in B6RO is larger in magnitude than the positive contribution of δ_0111_ and hence further decreases its overall δ_2PA_ value. This is the reason why B6RO has the lowest two-photon activity. In more detail, in the case of B6RO, the two rings are perpendicular to each other, which drastically hampers the charger transfer between the donor and acceptor groups. This is supported by the values of μ^01^ and μ^10^ (note that since we use a non-Hermitian formulation of coupled cluster theory, left and right transition moments are different). The values of μ^01^ and μ^10^ are 0.38 and 0.65 au, respectively, for B6RO, which is significantly smaller than the respective values in B5RO (1.88 and 3.31 au, respectively) and B7RO (1.09 and 1.93 au, respectively). The values of the other two dipole moments, *viz.*, μ^00^ and μ^11^ are also much smaller for B6RO as compared to those for B5RO and B7RO. This makes the δ_2PA_ value for B6RO much smaller as compared to the other two open systems. In open systems (B5RO → B7RO), the values of the product of the four involved transition/dipole moments are 167.2, 3.9, and 57.6 au, respectively, which follows the order of their δ_2PA_ values. However, in closed systems (B5RC → B7RC), the values are 200.3, 227.6, and 171.5 au, respectively. Thus, the order of δ_2PA_ values is controlled by the μ values in open systems but not in closed systems. Further analysis reveals that in B5RC, due to the larger value of μ^00^and μ^01^ as compared to that for B7RC, the negative contribution is also large, which decreases the overall value of δ_2PA_. This explains the δ_2PA_ order of B6RC > B7RC > B5RC. From the above discussion, we may conclude that in BP systems, when the rings are planar to each other, as in B5RC, the values of the ground-state dipole moment and transition dipole moment between *S*_0_ and *S*_1_ states increase, but it also causes an increase in the negative contribution from δ_0101_ and δ_0110_. This causes a detrimental effect on the overall δ_2PA_ value.

Analyzing the GFSM results in other planar B5RC-X systems, we noticed that for X = –NH, –O, and –S, different δ_0*fjk*_ terms are comparable to those in B5RC. This is because the involved transition dipole moments do not change significantly by these heteroatom-containing groups. However, for X = –NH and –O, the negative contribution of δ_0101_ + δ_0110_ is slightly larger than the positive contribution from δ_0111_, whereas for X = –S, the opposite is true. This is the reason why in comparison to B5RC, B5RC-NH and B5RC-O have slightly smaller δ_2PA_ values and B5RC-S have slightly larger δ_2PA_ values. Interestingly, for X = –BH and –BF, the involved transition dipole moments are decreased significantly. In B5RC-BH (B5RC-BF), the values of μ^00^, μ^01^, μ^10^, and μ^11^ are 3.36, 0.68, 1.12, and 5.91 au (3.39, 0.82, 1.37, and 6.04 au), respectively. In comparison to B5RC, a large decrease is caused in μ^01^ and μ^10^ of B5RC-BH and B5RC-BF. This is evidently due to the presence of the B atom and its empty p-orbital.

In TPs, when the rings are bridged by −CH_2_ unit(s), the transition dipole moments do not change significantly. In comparison to the open system, *i.e.*, T5RO, the closed ones have slightly smaller values for the *S*_1_ state dipole moment. However, when the rings are closed by –BH unit(s), we observed the same effect as observed in respective BP systems, *i.e.*, the transition dipole moments, in particular μ^01^ and μ^10^, decrease to a larger extent and hence δ_2PA_ values are also decreased drastically. For example, the values of μ^00^, μ^01^, μ^10^, and μ^11^ in T5RO are 3.25, 4.64, 2.64, and 10.11 au, respectively, whereas the same in T5RC-ABC (and T5RC-BH-ABC) are 3.28, 4.88, 2.81, and 8.89 au (3.65, 0.14, 0.23, and 6.54 au), respectively. In contrast to systems with the −CH_2_ bridge, the value of δ_2PA_ in systems with the –BH bridge is affected by the position of the bridge too. If we make all the rings planar to each other with –BH bridges, the transition moments μ^01^ and μ^10^ decrease to the largest extent; hence, T5RC-BH-ABC exhibits the minimum δ_2PA_ value. This indicates that the charge transfer between the donor and acceptor groups is largely hampered when the rings are made planar with the –BH unit. Among the other two systems (T5RC-BH-AB and T5RC-BH-BC), when rings A and B are made planar, the decrease in μ^01^ and μ^10^ is more than when rings B and C are made planar. This is because the –BH bridge also acts as an electron acceptor, so when the –BH bridge is close to donor-containing ring, the effective distance between the donor and acceptor groups is decreased than when the –BH bridge is close to the acceptor-containing ring. This decreases the transition dipole moments more in the former case than in the latter. Additionally, the dipole moment of the first excited state is much larger in T5RC-BH-BC (7.90 au) as compared to those in T5RC-BH-AB (5.75 au) and T5RC-BH-ABC (6.54 au). This explains the dependence of δ_2PA_ on the bridge position in the T5RC-BH systems.

## Summary

Various structural features of a molecule affect its two-photon absorption activity. In this work, we explored the effect of planarity of the chromophore and the presence of –BH unit(s) on 2PA properties of bi- and ter-phenyl systems. To achieve our goal, we have considered 11 bi-systems and 7 ter-phenyl systems having a D–π–A structure. The planar systems are made by bridging the rings at the *ortho*-positions by −CH_2_ and/or –BH units. To further study the effect of various bridge types, we considered creating a bridge using other heteroatoms/groups such as –O, –S, and –NH. Using the state-of-the-art RI-CC2 method with the cc-pVDZ basis set, we calculated the two-photon spectra in all of the systems. Furthermore, to discuss and explain the variation of 2PA activity in these systems, we performed two-state model-based analyses. The work clearly demonstrates that in bi- and ter-phenyl systems, when the rings are made planar *via* the −CH_2_ bridge, the 2PA activity increases slightly. However, when the bridge(s) are replaced by –BH bridge(s), a drastic decrease in 2PA activity is observed. In the ter-phenyl systems, since there are two bridges possible, the 2PA activity (in the case of –BH bridges) is found to be dependent on their positions too. In comparison to the open systems (no bridge), the 2PA activity decreases drastically when the donor-containing ring is connected with the middle ring *via* –BH bridge than when the bridge is between the middle ring and the acceptor-containing ring. An in-depth 2SM analysis reveals that in both bi- and ter-phenyl systems, due to the presence of the –B atom and its empty p-orbital, the *S*_0_ → *S*_1_ transition is drastically affected, resulting in a large drop in the value of the corresponding transition dipole moments (μ^01^ and μ^10^). This is further supported by the large decrease in the *d*_CT_ values due to –BH bridging. In ter-phenyl, *d*_CT_ values depend upon the position of the –BH bridge, which is consistent with our 2PA observations. In conclusion, as far as 2PA activity is concerned, this work suggests avoiding –BH bridge(s) to enforce planarity in bi- and ter-phenyl systems; however, one may use −CH_2_ bridge(s) that enhances the 2PA.

## Supporting Information Available

Optimized coordinates; orbitals involved in the *S*_0_ → *S*_1_ transition, and transition dipole moment vectors involved in the two-state model in all compounds (PDF)
