# Supplementary material for: Chromophore Planarity, –BH Bridge Effect, and Two-Photon Activity: Bi- and Ter-Phenyl Derivatives as a Case Study
Source: J Phys Chem A. 2023 Sep 18;127(38):7928–36. doi: 10.1021/acs.jpca.3c04288 (PMC10544031; doi:10.1021/acs.jpca.3c04288)
Supplement: Supplementary file 1 — jp3c04288_si_001.pdf [file jp3c04288_si_001.pdf]

Supplementary Information File

Chromophore planarity, -BH bridge, and two-photon activity: Bi- and Ter-Phenyl systems as a case study

Swati Singh Rajput,[†] Robert Zaleśny,*,[‡] and Md Mehboob Alam*,[†]

[†]Department of Chemistry, Indian Institute of Technology Bhilai, GEC campus, Sejbahar, Raipur, CG-492015 India

[‡]Faculty of Chemistry, Wroclaw University of Science and Technology, Wyb. Wyspiańskiego 27, PL–50370 Wroclaw, Poland

E-mail: robert.zalesny@pwr.edu.pl, mehboob@iitbhiali.ac.in

Content

1. Orbital diagram
2. Optimized coordinates
3. Transition dipole moment vectors

1. **Orbital Diagram**:

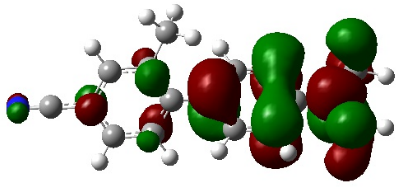

B5RO (HOMO)

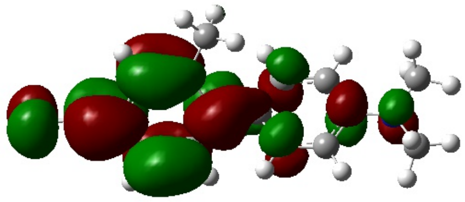

B5RO (LUMO)

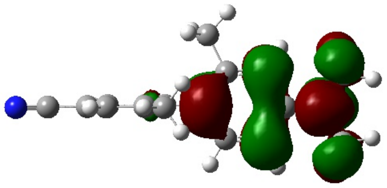

B6RO (HOMO)

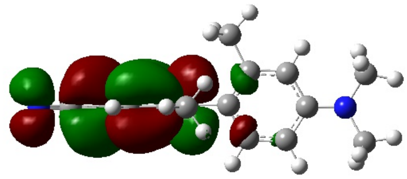

B6RO (LUMO)

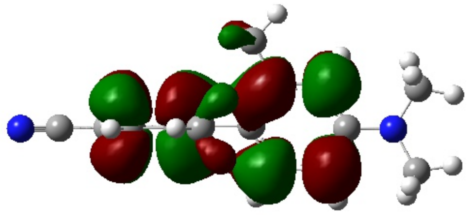

B6RO (LUMO+1)

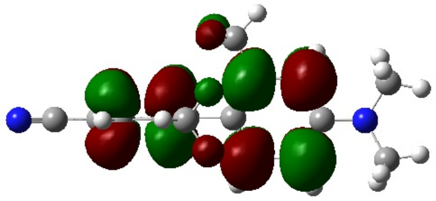

B6RO (LUMO+2)

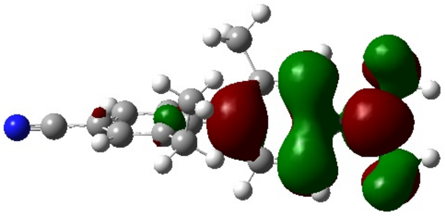

B7RO (HOMO)

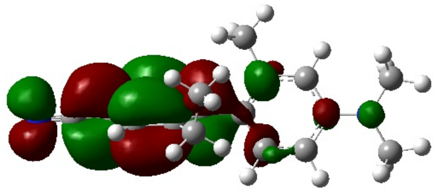

B7RO (LUMO)

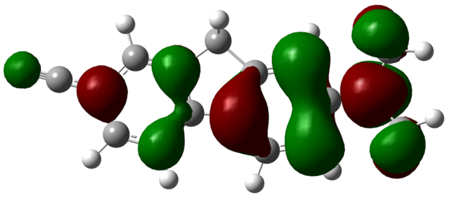

B5RC (HOMO)

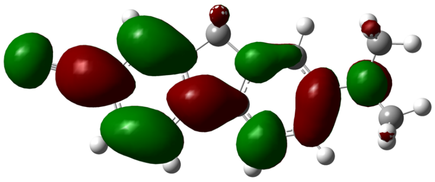

B5RC (LUMO)

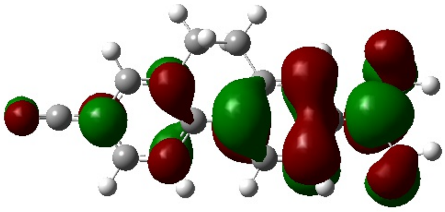

B6RC (HOMO)

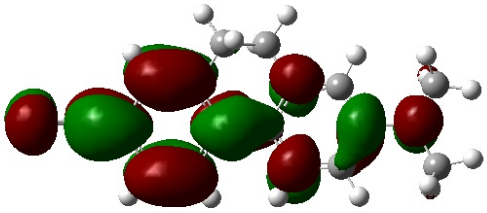

B6RC (LUMO)

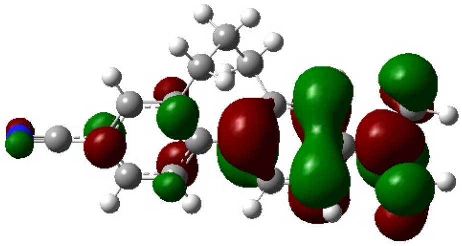

B7RC (HOMO)

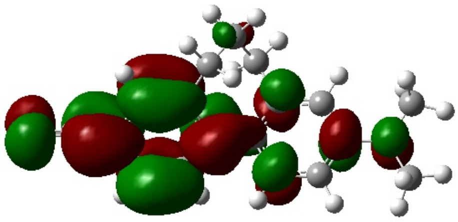

B7RC (LUMO)

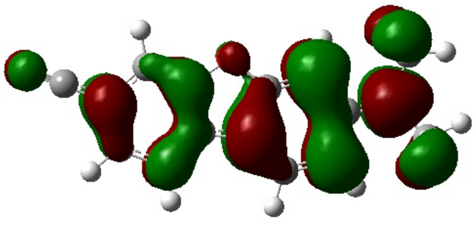

B5RC-O (HOMO)

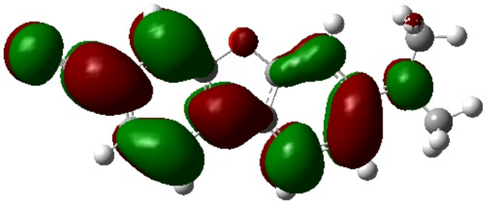

B5RC-O (LUMO)

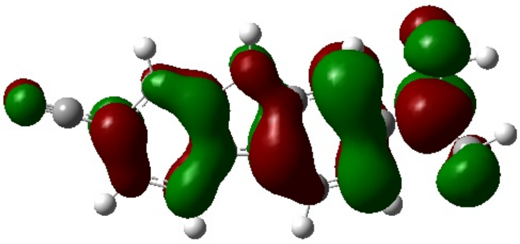

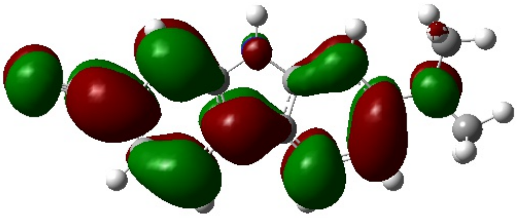

B5RC-NH (HOMO)

B5RC-NH (LUMO)

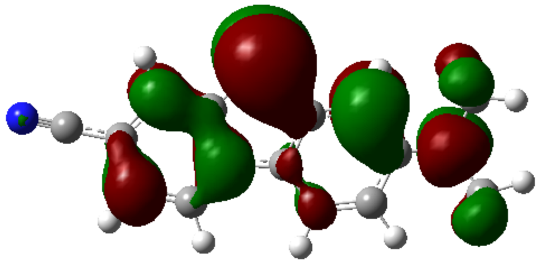

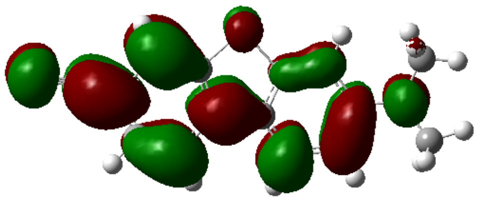

B5RC-S (HOMO)

B5RC-S (LUMO)

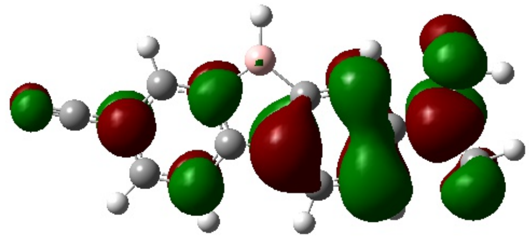

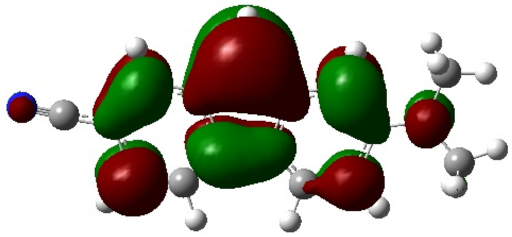

B5RC-BH (HOMO)

B5RC-BH (LUMO)

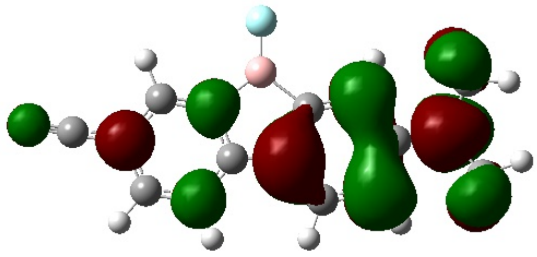

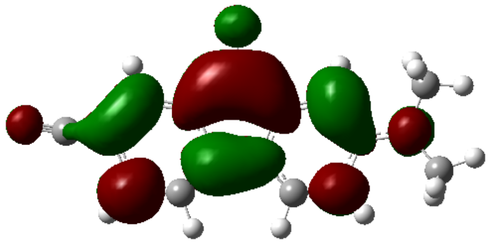

B5RC-BF (HOMO)

B5RC-BF (LUMO)

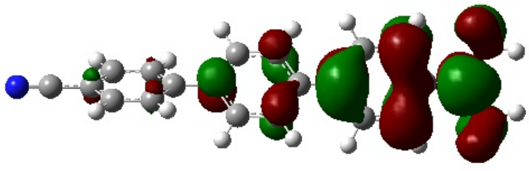

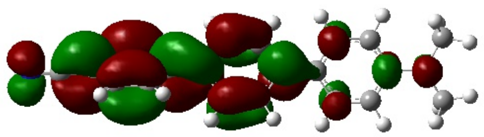

T5RO (HOMO)

T5RO (LUMO)

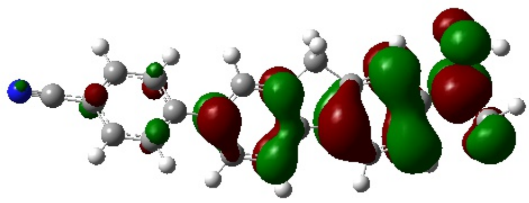

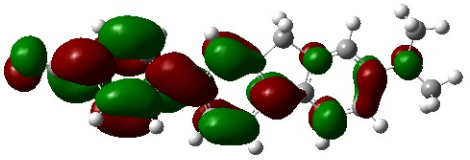

T5RC-AB (HOMO)

T5RC-AB (LUMO)

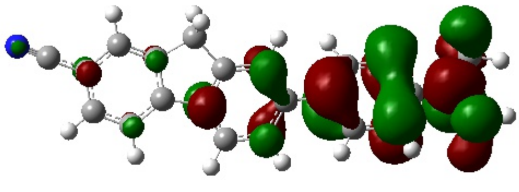

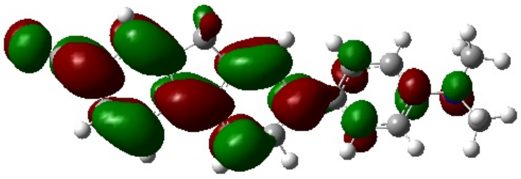

T5RC-BC (HOMO) T5RC-BC (LUMO)

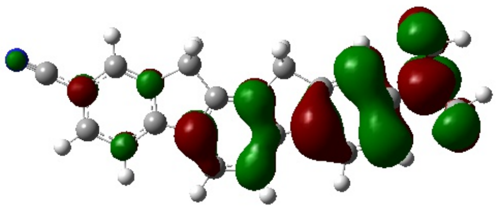

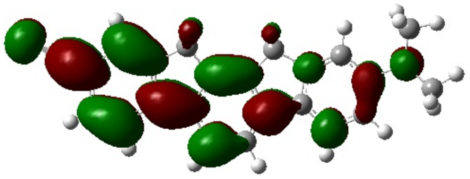

T5RC-ABC (HOMO) T5RC-ABC (LUMO)

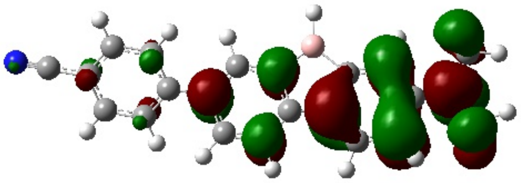

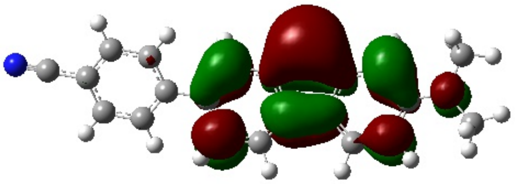

T5RC-BH-AB (HOMO) T5RC-BH-AB (LUMO 2)

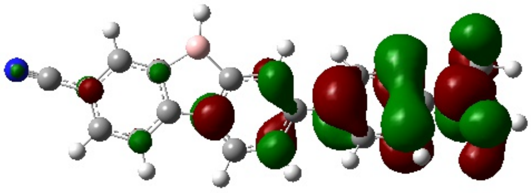

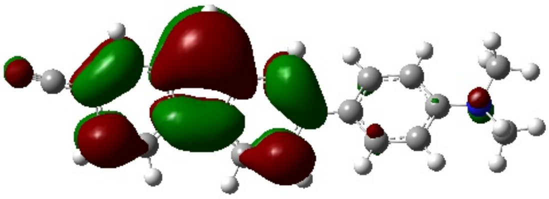

T5RC-BH-BC (HOMO) T5RC-BH-BC (LUMO)

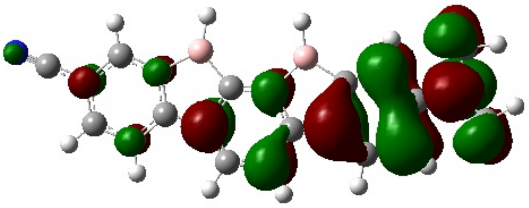

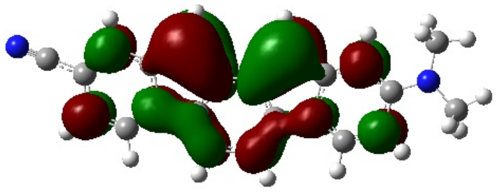

T5RC-BH-ABC (HOMO) T5RC-BH-ABC (LUMO)

2. **Optimized Coordinates:** This includes the optimized cartesian coordinates for all the BP and TP system considered in this work, that are optimized at B3LYP/6-311+G(d,p) level of theory using Gaussian 16 program.

B5RO			
C	-1.221377	-0.100924	-0.052352
C	-1.998980	1.001468	0.374782
C	-3.389954	0.896619	0.340359
C	-4.030787	-0.267489	-0.101411
C	-3.261176	-1.363010	-0.511661
C	-1.878718	-1.266694	-0.480675
C	0.264861	-0.087835	-0.049696
C	1.008303	0.907489	-0.696756
C	2.395890	0.886751	-0.725749
C	3.125285	-0.148086	-0.098596
C	2.376929	-1.143344	0.568014
C	0.989007	-1.111500	0.576261
H	-3.994764	1.730582	0.677953
H	-3.745470	-2.268806	-0.854793
H	-1.282008	-2.106847	-0.816437
H	0.494742	1.710588	-1.212769
H	2.910972	1.679975	-1.249273
H	2.876316	-1.948403	1.088624
H	0.454758	-1.893532	1.105406
C	-5.459153	-0.336138	-0.122963
N	-6.613930	-0.391592	-0.140534
N	4.509779	-0.190816	-0.146289
C	5.225700	-1.145523	0.685099
H	6.292374	-1.064798	0.479910
H	5.067347	-0.975634	1.760229
H	4.924223	-2.170758	0.453507
C	5.245199	0.961577	-0.642905
H	6.309784	0.731392	-0.639306
H	4.963737	1.192953	-1.673944
H	5.084975	1.862614	-0.032870
C	-1.383002	2.276420	0.904651
H	-0.473089	2.075853	1.472652
H	-1.114586	2.964085	0.096595
H	-2.088435	2.798895	1.553871

B6RO			
C	-1.243989	-0.120002	-0.163422
C	-1.976420	-0.157598	1.042950
C	-3.369942	-0.175545	0.985429
C	-4.048624	-0.158863	-0.240781

C	-3.321056	-0.126668	-1.435697
C	-1.933002	-0.107523	-1.382234
C	0.252133	-0.112197	-0.164368
C	0.990391	1.085346	-0.119606
C	2.384470	1.034455	-0.127799
C	3.099561	-0.180576	-0.192517
C	2.342261	-1.369067	-0.224993
C	0.954082	-1.318980	-0.216214
H	-3.944928	-0.205650	1.903833
H	-3.839538	-0.117506	-2.386425
H	-1.363219	-0.084341	-2.304362
H	2.920911	1.972807	-0.088021
H	2.826204	-2.334801	-0.260024
H	0.398866	-2.251019	-0.247504
N	4.488401	-0.201707	-0.238111
C	5.184240	-1.465811	-0.056568
H	6.255367	-1.299997	-0.166505
H	5.003538	-1.914689	0.931748
H	4.885948	-2.189846	-0.819314
C	5.230149	1.016052	0.047347
H	6.295078	0.817369	-0.067961
H	4.968068	1.809604	-0.657532
H	5.057541	1.392184	1.066978
C	-5.479364	-0.177335	-0.266365
N	-6.635120	-0.191639	-0.286507
C	0.297591	2.428146	-0.072450
H	-0.294769	2.602237	-0.975784
H	-0.392728	2.499183	0.773262
H	1.021954	3.239888	0.015464
C	-1.278827	-0.190540	2.381636
H	-0.659205	-1.085822	2.482291
H	-0.611831	0.666649	2.507201
H	-2.002140	-0.182600	3.198890

B7RO			
C	1.167887	-0.247397	-0.208364
C	1.970168	0.914798	-0.169547
C	3.358726	0.775775	-0.231933
C	3.966876	-0.481356	-0.326752
C	3.170219	-1.632629	-0.363920
C	1.790617	-1.500790	-0.306039
C	-0.326289	-0.186421	-0.178632
C	-1.072503	-0.590412	0.945144
C	-2.466620	-0.535823	0.901370
C	-3.175596	-0.089866	-0.233465
C	-2.411972	0.286830	-1.357784
C	-1.025776	0.241057	-1.312165
H	3.984951	1.660746	-0.207022
H	3.631127	-2.609112	-0.446855
H	1.170206	-2.388645	-0.352566

H	-3.007773	-0.849087	1.783949
H	-2.890589	0.613929	-2.269924
H	-0.467198	0.533202	-2.195606
C	5.392433	-0.585780	-0.391970
N	6.544273	-0.670386	-0.443128
N	-4.562578	-0.012033	-0.237454
C	-5.315503	-0.637661	0.837917
H	-6.376986	-0.445930	0.685311
H	-5.164823	-1.726488	0.887010
H	-5.042767	-0.211838	1.807271
C	-5.257067	0.217470	-1.494582
H	-6.326077	0.295898	-1.299812
H	-4.938467	1.158016	-1.951570
H	-5.097138	-0.590210	-2.224478
C	1.386369	2.305469	-0.015857
H	0.437441	2.375497	-0.550013
H	2.066464	3.023661	-0.483927
C	1.164722	2.705170	1.453623
H	0.775625	3.724964	1.519075
H	0.444869	2.040890	1.937385
H	2.099252	2.662100	2.019790
C	-0.400308	-1.092405	2.203893
H	0.449360	-0.466869	2.487365
H	-1.103263	-1.111467	3.038980
H	-0.014375	-2.108498	2.074101

**B5RC**			
C	-1.259012	0.595451	0.010650
C	-1.691644	-0.749511	0.010607
C	-3.037531	-1.060921	-0.001870
C	-3.979689	-0.013477	-0.014659
C	-3.550701	1.324982	-0.014528
C	-2.195427	1.632788	-0.001920
C	0.200969	0.614603	0.025267
C	0.674989	-0.710270	0.034406
C	2.028806	-0.995755	0.044471
C	2.972147	0.060753	0.055448
C	2.481255	1.391685	0.032730
C	1.120589	1.664006	0.021531
H	-3.380271	-2.089381	-0.002103
H	-4.290487	2.116106	-0.024309
H	-1.878948	2.669583	-0.001680
H	2.356883	-2.026437	0.043822
H	3.172769	2.222016	0.023146
H	0.788635	2.696642	0.005253
C	-5.376440	-0.313503	-0.027732
N	-6.506801	-0.559277	-0.038341
N	4.332254	-0.196049	0.096209
C	5.280365	0.891312	-0.091135

H	6.291978	0.500419	0.007575
H	5.189671	1.365906	-1.078587
H	5.150835	1.663564	0.672524
C	4.812812	-1.558244	-0.071926
H	5.897396	-1.567035	0.025311
H	4.408284	-2.217199	0.701946
H	4.551776	-1.980596	-1.052998
C	-0.492972	-1.675529	0.025355
H	-0.492823	-2.325592	0.907463
H	-0.474947	-2.330937	-0.852607

B6RC			
C	1.234637	-0.344791	-0.001171
C	1.945994	0.858734	-0.217841
C	3.334878	0.857761	-0.198090
C	4.053004	-0.324620	0.035044
C	3.353531	-1.518173	0.262401

C	1.967574	-1.517685	0.248609
C	-0.238931	-0.324328	-0.037500
C	-0.925121	0.896513	0.132700
C	-2.313912	0.934930	0.128942
C	-3.093115	-0.228425	-0.057137
C	-2.397401	-1.446383	-0.231847
C	-1.012944	-1.478763	-0.224692
H	3.876438	1.781981	-0.366221
H	3.899842	-2.432236	0.460045
H	1.446620	-2.444560	0.454146
H	-2.795512	1.893186	0.271825
H	-2.936274	-2.370557	-0.385538
H	-0.526569	-2.433562	-0.387489
N	-4.474901	-0.174954	-0.088290
C	-5.157766	1.055723	0.278419
H	-6.231302	0.914508	0.162066
H	-4.960372	1.354473	1.317862
H	-4.861774	1.880798	-0.375965
C	-5.243743	-1.409960	-0.095182
H	-6.303549	-1.167939	-0.160174
H	-4.993212	-2.024960	-0.964234
H	-5.082793	-2.012761	0.809903
C	5.481415	-0.307670	0.053018
N	6.637923	-0.293248	0.066527
C	-0.117695	2.156407	0.350163
H	0.148027	2.237752	1.412878
H	-0.715459	3.037854	0.104494
C	1.165011	2.124067	-0.487003
H	1.784341	3.001090	-0.282478
H	0.897887	2.162336	-1.551792

**B7RC**			
C	1.208723	-0.430510	0.040541
C	1.953712	0.644815	-0.501380
C	3.344606	0.585456	-0.487838
C	4.020828	-0.522102	0.043801
C	3.284992	-1.593466	0.566539
C	1.898902	-1.536742	0.560653
C	-0.273435	-0.403095	0.047186
C	-0.988213	0.741301	0.464064
C	-2.380344	0.730393	0.460435
C	-3.126742	-0.398428	0.055964
C	-2.398674	-1.532209	-0.364315
C	-1.011903	-1.523256	-0.354536
H	3.920333	1.401288	-0.910313
H	3.800242	-2.451375	0.980631
H	1.333264	-2.356638	0.988037
H	-2.891666	1.621787	0.798678
H	-2.909103	-2.421065	-0.707069
H	-0.485786	-2.407698	-0.697993

C	5.450163	-0.556834	0.045936
N	6.606232	-0.584027	0.047502
N	-4.512471	-0.396457	0.085393
C	-5.242758	-1.496511	-0.524470
H	-6.309337	-1.348862	-0.360054
H	-5.068040	-1.573889	-1.607621
H	-4.969342	-2.450687	-0.065812
C	-5.224898	0.852501	0.303827
H	-6.295382	0.652195	0.319323
H	-4.960293	1.293629	1.268963
H	-5.024871	1.597444	-0.480097
C	1.240675	1.816025	-1.138010
H	0.502852	1.419897	-1.843643
H	1.950733	2.405722	-1.723628
C	0.512219	2.736082	-0.136540
H	-0.186256	3.367722	-0.695492
H	1.230272	3.408197	0.345215
C	-0.241808	1.959429	0.963391
H	-0.934033	2.634857	1.472798
H	0.483827	1.633758	1.716338

**B5RC-NH**			
C	1.250824	0.691889	-0.010897
C	1.634895	-0.678213	-0.009566
C	2.964763	-1.072104	0.003059
C	3.941573	-0.064776	0.015043
C	3.580183	1.299440	0.014220
C	2.244844	1.676080	0.001336
C	-0.192063	0.715680	-0.026088
C	-0.631020	-0.630234	-0.033529
C	-1.975442	-0.988058	-0.042850
C	-2.944400	0.035187	-0.057326
C	-2.505902	1.392127	-0.037449
C	-1.162859	1.722941	-0.025064
H	3.254675	-2.115771	0.003995
H	4.360643	2.049533	0.023657
H	1.980613	2.727271	0.000467
H	-2.257045	-2.031433	-0.038899
H	-3.233025	2.190695	-0.031822
H	-0.873598	2.768039	-0.010987
C	5.323312	-0.429131	0.028327
N	6.441550	-0.725055	0.039140
N	-4.296348	-0.265774	-0.099792
C	-5.282606	0.783103	0.113744
H	-6.279333	0.355016	0.017302
H	-5.201268	1.244497	1.107964
H	-5.190867	1.571341	-0.638524
C	-4.726436	-1.645359	0.053484
H	-5.809427	-1.695067	-0.049064

H	-4.293233	-2.279971	-0.725557
H	-4.453117	-2.067810	1.031646
N	0.485042	-1.453250	-0.022294
H	0.465277	-2.459215	-0.028231

B5RC-O			
C	1.250200	0.754311	-0.008104
C	1.594492	-0.612967	-0.009381
C	2.893575	-1.074122	0.000304
C	3.908026	-0.100910	0.012055
C	3.595054	1.274216	0.013640
C	2.275285	1.706086	0.003634
C	-0.193837	0.785210	-0.020572
C	-0.604284	-0.557212	-0.028508
C	-1.920756	-0.972982	-0.036326
C	-2.921965	0.024285	-0.046351
C	-2.521945	1.392818	-0.027331
C	-1.189214	1.767984	-0.017477
H	3.123753	-2.131202	-0.000981
H	4.403313	1.994445	0.022741
H	2.050881	2.766063	0.004781
H	-2.150423	-2.027691	-0.034092
H	-3.273325	2.168730	-0.020028
H	-0.931344	2.820871	-0.003937
C	5.276771	-0.511860	0.022573
N	6.385256	-0.841412	0.031176
N	-4.259543	-0.318648	-0.081026
C	-5.281887	0.703026	0.089218
H	-6.262962	0.238233	0.006859
H	-5.219489	1.201212	1.066329
H	-5.213218	1.467114	-0.690776
C	-4.647470	-1.714706	0.045118
H	-5.729436	-1.793660	-0.046354
H	-4.203269	-2.319998	-0.750978
H	-4.351041	-2.146792	1.011123
O	0.475537	-1.411995	-0.021338

B5RC-S			
C	1.219665	0.627320	-0.011623
C	1.749156	-0.684983	-0.003846
C	3.116363	-0.924502	0.008776
C	3.987378	0.173129	0.013985
C	3.478471	1.487884	0.006367
C	2.111884	1.710097	-0.006338
C	-0.226307	0.629983	-0.024238
C	-0.770813	-0.670191	-0.026727
C	-2.139296	-0.911843	-0.033246
C	-3.034478	0.177620	-0.049138
C	-2.488576	1.491984	-0.034494

C	-1.123804	1.706912	-0.025399
H	3.513456	-1.931856	0.014617
H	4.168881	2.321844	0.010403
H	1.734401	2.726083	-0.012469
H	-2.502132	-1.929316	-0.026302
H	-3.145891	2.349287	-0.029707
H	-0.751415	2.725475	-0.015195
C	5.399910	-0.043302	0.026936
N	6.543336	-0.216068	0.037458
N	-4.401567	-0.021634	-0.085249
C	-5.307219	1.103754	0.092813
H	-6.332844	0.749457	0.003159
H	-5.194618	1.583815	1.074703
H	-5.153168	1.862368	-0.680026
C	-4.936722	-1.366594	0.055751
H	-6.020686	-1.330390	-0.039652
H	-4.558239	-2.025136	-0.731852
H	-4.691488	-1.817079	1.027904
S	0.482981	-1.916516	-0.012407

B5RC-BF			
C	-1.243979	-0.679197	-0.008951
C	-1.743400	0.652497	-0.005856
C	-3.108176	0.886111	0.004764
C	-4.000810	-0.205618	0.012523
C	-3.498552	-1.515570	0.009245
C	-2.124745	-1.757215	-0.001467
C	0.236546	-0.684026	-0.020696
C	0.748096	0.636592	-0.026715
C	2.112578	0.872481	-0.035554
C	3.031912	-0.210952	-0.046500
C	2.497100	-1.520252	-0.026218
C	1.121698	-1.753459	-0.017593
H	-3.501761	1.896408	0.007298
H	-4.194480	-2.346119	0.015082
H	-1.763802	-2.779454	-0.004121
H	2.471780	1.893022	-0.033323
H	3.160535	-2.373645	-0.016140
H	0.766940	-2.778788	-0.003804
C	-5.412169	0.017287	0.023505
N	-6.554285	0.199047	0.032447
N	4.395471	0.008298	-0.082057
C	5.312702	-1.110614	0.064920
H	6.334918	-0.746906	-0.025249
H	5.211020	-1.612468	1.037483
H	5.156495	-1.855184	-0.721727
C	4.918146	1.358017	0.072415
H	6.002686	1.330396	-0.019389
H	4.538097	2.021681	-0.709706
H	4.667112	1.797044	1.047795

B	-0.490673	1.584799	-0.015919
F	-0.485129	2.918012	-0.014919

B5RC-BH			
C	1.278475	0.493323	-0.009422
C	1.751892	-0.851652	-0.010466
C	3.115443	-1.101310	0.000577
C	4.026653	-0.023638	0.012688
C	3.545470	1.293365	0.013454
C	2.174154	1.556967	0.002451
C	-0.199625	0.511750	-0.022538
C	-0.708920	-0.812835	-0.033316
C	-2.076628	-1.039646	-0.044709
C	-2.995138	0.046247	-0.053647
C	-2.458341	1.352573	-0.026923
C	-1.079929	1.582307	-0.016212
H	3.495238	-2.116979	0.000146
H	4.254280	2.113028	0.022656
H	1.829948	2.585155	0.003001
H	-2.440145	-2.058748	-0.045715
H	-3.119074	2.208037	-0.013686
H	-0.723329	2.607136	0.002110
C	5.434013	-0.269923	0.024043
N	6.572834	-0.471972	0.033278
N	-4.360610	-0.171487	-0.095103
C	-5.273337	0.948037	0.073348
H	-6.297100	0.590453	-0.024748
H	-5.170047	1.432041	1.055125
H	-5.113277	1.705940	-0.699334
C	-4.885864	-1.517862	0.078697
H	-5.969319	-1.490989	-0.026766
H	-4.498269	-2.194983	-0.687512
H	-4.646540	-1.940620	1.064560
B	0.506284	-1.792087	-0.023880
H	0.490743	-2.981046	-0.025806

T5RO			
C	-0.838978	0.010538	0.022826
C	-0.103640	1.194482	0.194947
C	1.285411	1.191473	0.187538
C	2.008408	0.003040	0.007046
C	1.277129	-1.181649	-0.165423
C	-0.111828	-1.177331	-0.157561
C	-2.318434	0.015222	0.031376
C	-3.054420	1.072182	-0.522940
C	-4.442044	1.084136	-0.523182
C	-5.182180	0.028699	0.054252
C	-4.442985	-1.043817	0.600635
C	-3.055482	-1.040255	0.587193

H	1.801259	-2.122451	-0.291429
H	-0.642429	-2.110070	-0.310680
H	-4.948809	1.918452	-0.987631
H	-4.950607	-1.884573	1.052429
H	-2.534456	-1.874283	1.044365
N	-6.568474	0.049466	0.094023
C	-7.290370	-1.156943	0.466950
H	-8.358420	-0.943042	0.474281
H	-7.110047	-1.991492	-0.226538
H	-7.015276	-1.484098	1.473337
C	-7.290176	1.059677	-0.663179
H	-8.356621	0.955381	-0.466975
H	-6.996765	2.065703	-0.351672
H	-7.128895	0.975371	-1.748182
C	3.489468	-0.000908	-0.001037
C	4.206893	-0.835270	-0.874539
C	4.220704	0.829593	0.864666
C	5.594178	-0.844130	-0.886328
H	3.669623	-1.467336	-1.571363
C	5.608063	0.831252	0.861335
C	6.309307	-0.008282	-0.016352
H	6.131167	-1.487141	-1.572917
H	6.155826	1.471410	1.542054
C	7.739010	-0.012055	-0.024126
N	8.895135	-0.015108	-0.030397
H	3.694347	1.464444	1.567266
H	1.815732	2.129570	0.307683
H	-0.627717	2.129941	0.353932
H	-2.532727	1.895793	-0.997927

T5RC-AB			
C	-0.800533	0.764702	-0.123719
C	-0.271438	-0.533579	0.030453
C	1.095867	-0.743739	0.062645
C	1.979143	0.347089	-0.053718
C	1.439681	1.636363	-0.205274
C	0.065239	1.852934	-0.243760
C	-2.260305	0.678433	-0.120264
C	-2.636451	-0.667607	0.040638
C	-3.966208	-1.048950	0.085374
C	-4.984950	-0.073892	-0.042768
C	-4.592980	1.280332	-0.189437
C	-3.254678	1.649824	-0.231360
H	2.109784	2.480183	-0.324203
H	-0.318480	2.858774	-0.374856
H	-4.217000	-2.092808	0.218150
H	-5.342890	2.054089	-0.271686
H	-2.999787	2.698065	-0.346268
N	-6.325700	-0.433399	-0.036514
C	-7.345777	0.598918	0.060804

H	-8.328452	0.131192	0.016427
H	-7.279814	1.175042	0.995413
H	-7.278298	1.298481	-0.776830
C	-6.700623	-1.787969	0.336037
H	-7.781140	-1.893979	0.248927
H	-6.244708	-2.520683	-0.335713
H	-6.411201	-2.041105	1.366765
C	3.444978	0.139692	-0.019190
C	4.293716	1.059143	0.620941
C	4.033001	-0.984019	-0.624921
C	5.667575	0.870465	0.655577
H	3.867279	1.920824	1.120222
C	5.405177	-1.185576	-0.594792
C	6.237908	-0.257283	0.047075
H	6.304938	1.585387	1.161492
H	5.841143	-2.052919	-1.075372
C	7.652745	-0.458676	0.080962
N	8.797101	-0.621742	0.108432
C	-1.400206	-1.537538	0.153054
H	-1.360654	-2.294877	-0.638081
H	-1.365046	-2.074118	1.108076
H	1.491828	-1.743598	0.207217
H	3.407405	-1.696372	-1.149257

**T5RC-BC**			
C	-0.870026	0.363849	-0.100988
C	-0.019439	-0.733019	0.131181
C	1.355736	-0.564939	0.100553
C	1.921334	0.698616	-0.155224
C	1.091780	1.797299	-0.384770
C	-0.286799	1.620638	-0.358652
C	-2.341122	0.202784	-0.076684
C	-2.963816	-0.954632	-0.565651
C	-4.341997	-1.115375	-0.546385
C	-5.188387	-0.107579	-0.033585
C	-4.562644	1.051616	0.476060
C	-3.182853	1.196039	0.444367
H	1.507269	2.777162	-0.593798
H	-0.931519	2.466480	-0.566782
H	-4.758726	-2.028971	-0.946878
H	-5.152510	1.845522	0.912418
H	-2.749684	2.095359	0.868214
N	-6.568756	-0.245174	-0.041683
C	-7.386084	0.710593	0.688675
H	-8.437270	0.476790	0.524329
H	-7.194253	0.695649	1.771900
H	-7.216464	1.727897	0.326108
C	-7.154678	-1.542641	-0.339767
H	-8.240130	-1.451585	-0.332657
H	-6.861783	-1.886342	-1.335547

H	-6.868103	-2.315831	0.388294
C	3.379154	0.576894	-0.118077
C	4.385405	1.526689	-0.309077
C	3.711355	-0.764982	0.166763
C	5.715116	1.134067	-0.215571
H	4.142619	2.560087	-0.528262
C	5.032868	-1.161078	0.261430
C	6.045443	-0.202596	0.068255
H	6.510145	1.855093	-0.360664
H	5.301578	-2.188224	0.479861
C	7.418027	-0.590735	0.161270
N	8.527936	-0.906769	0.236898
C	2.450170	-1.586485	0.328622
H	2.403663	-2.406395	-0.396912
H	2.386044	-2.040059	1.324146
H	-2.359662	-1.745676	-0.996247
H	-0.446743	-1.703504	0.359500

**T5RC-ABC**			
C	-0.851436	1.083196	-0.016349
C	-0.188017	-0.165393	-0.023232
C	1.192753	-0.203122	-0.014059
C	1.923341	1.007217	0.002011
C	1.267354	2.241666	0.008702
C	-0.125315	2.279862	-0.000497
C	-2.293680	0.838894	-0.028887
C	-2.523743	-0.548861	-0.043657
C	-3.804511	-1.073051	-0.053330
C	-4.922757	-0.204310	-0.059071
C	-4.678422	1.191349	-0.029345
C	-3.387480	1.704108	-0.018520
H	1.833097	3.166865	0.020751
H	-0.639280	3.234644	0.004286
H	-3.941150	-2.146148	-0.056970
H	-5.507155	1.884790	-0.014484
H	-3.247221	2.779658	0.002902
N	-6.217510	-0.702984	-0.104548
C	-7.340625	0.193232	0.121504
H	-8.268300	-0.367697	0.015713
H	-7.325578	0.649506	1.122181
H	-7.357821	0.996886	-0.619787
C	-6.442381	-2.126660	0.086643
H	-7.506251	-2.335349	-0.018286
H	-5.917529	-2.712798	-0.672962
H	-6.117860	-2.479544	1.076858
C	3.350431	0.685592	0.008758
C	4.477444	1.511587	0.023833

C	3.499909	-0.718246	-0.002983
C	5.741965	0.935650	0.027182
H	4.377240	2.590826	0.032899
C	4.755413	-1.296765	0.000297
C	5.889373	-0.462362	0.015545
H	6.627094	1.559757	0.038784
H	4.882372	-2.373276	-0.008590
C	7.196568	-1.039957	0.019273
N	8.253442	-1.509853	0.022299
C	2.139645	-1.383405	-0.018256
H	2.006452	-2.014915	-0.904440
H	1.994491	-2.030833	0.854466
C	-1.201217	-1.290240	-0.040189
H	-1.089408	-1.928696	-0.924528
H	-1.102394	-1.943698	0.834706

**T5RC-BH-AB**			
C	-0.807319	0.644139	0.069364
C	-0.253670	-0.655418	-0.109061
C	1.125055	-0.820180	-0.122374
C	1.989702	0.284090	0.033539
C	1.415478	1.551135	0.205162
C	0.029116	1.740892	0.225755
C	-2.286263	0.575963	0.049463
C	-2.713703	-0.762713	-0.148681
C	-4.065482	-1.068578	-0.201234
C	-5.049061	-0.051046	-0.066334
C	-4.593672	1.267933	0.146257
C	-3.230893	1.578264	0.198154
H	1.546294	-1.807738	-0.281322
H	2.062376	2.409095	0.352841
H	-0.369797	2.739055	0.371834
H	-4.365195	-2.098012	-0.347301
H	-5.305337	2.071524	0.274630
H	-2.938109	2.610490	0.361861
N	-6.401449	-0.344548	-0.151405
C	-7.378324	0.679059	0.182500
H	-8.379365	0.281778	0.020672
H	-7.303779	1.011169	1.228535
H	-7.264257	1.555426	-0.462102
C	-6.839726	-1.732165	-0.138430
H	-7.922968	-1.761683	-0.247456
H	-6.411135	-2.288944	-0.976144
H	-6.572918	-2.252712	0.792610
B	-1.438956	-1.656521	-0.266330
H	-1.383395	-2.833259	-0.435317
C	3.460430	0.110745	0.017667
C	4.298064	1.061324	-0.590162
C	4.062229	-1.012590	0.609806

C	5.676284	0.903975	-0.604838
H	3.861061	1.922936	-1.080635
C	5.439000	-1.182801	0.599336
H	3.443862	-1.750411	1.106797
C	6.261060	-0.222911	-0.008800
H	6.305942	1.642623	-1.085648
H	5.886182	-2.050454	1.068841
C	7.680681	-0.391525	-0.021701
N	8.828792	-0.527428	-0.031932

**T5RC-BC**			
C	-0.862889	0.299464	-0.091449
C	-0.025124	-0.811378	0.153818
C	1.358560	-0.685929	0.132990
C	1.944173	0.580662	-0.134557
C	1.142202	1.685011	-0.374787
C	-0.249658	1.533449	-0.355597
C	-2.335964	0.166711	-0.068372
C	-2.977504	-0.989104	-0.537071
C	-4.358216	-1.123198	-0.522422
C	-5.188105	-0.089605	-0.033809
C	-4.543269	1.067568	0.457286
C	-3.161161	1.185258	0.430197
H	1.568870	2.659264	-0.589357
H	-0.871990	2.393801	-0.574512
H	-4.790117	-2.036963	-0.905954
H	-5.120359	1.879848	0.876482
H	-2.713532	2.083772	0.840773
N	-6.569536	-0.202008	-0.045427
C	-7.374365	0.786763	0.654393
H	-8.428435	0.566524	0.490336
H	-7.188567	0.796678	1.738540
H	-7.185238	1.791245	0.266296
C	-7.179798	-1.491244	-0.330871
H	-8.263186	-1.378804	-0.329695
H	-6.889134	-1.852831	-1.321025
H	-6.911678	-2.260480	0.407992
C	3.423308	0.477798	-0.101659
C	4.376707	1.466151	-0.310565
C	3.812819	-0.859031	0.193745
C	5.731096	1.133267	-0.227905
H	4.092007	2.487709	-0.536137
C	5.159375	-1.178982	0.274482
C	6.131388	-0.178037	0.062357
H	6.487183	1.892544	-0.388742
H	5.479449	-2.190364	0.498697
C	7.522395	-0.497731	0.142654
N	8.646917	-0.759077	0.208240
H	-2.386633	-1.800663	-0.947278
H	-0.473586	-1.771822	0.385043

B	2.510163	-1.706843	0.369529
H	2.426652	-2.864822	0.624066

T5RC-ABC			
C	-0.845166	-0.913870	0.014663
C	-0.172227	0.352068	0.020072
C	1.216864	0.384973	0.012603
C	1.946123	-0.846112	-0.000020
C	1.285629	-2.062694	-0.005005
C	-0.124248	-2.097894	0.002356
C	-2.309599	-0.706898	0.024571
C	-2.615338	0.679700	0.038256
C	-3.935389	1.109996	0.048125
C	-5.005843	0.177101	0.053287
C	-4.670517	-1.195506	0.022497
C	-3.343304	-1.631333	0.013180
H	1.837017	-2.997501	-0.014407
H	-0.629440	-3.058086	-0.001303
H	-4.139617	2.172556	0.051367
H	-5.452682	-1.941691	0.005125
H	-3.146383	-2.698401	-0.008210
N	-6.325443	0.596357	0.096147
C	-7.393718	-0.371472	-0.094253
H	-8.353182	0.133343	0.008066
H	-7.359902	-0.849862	-1.083961
H	-7.352898	-1.157100	0.665949
C	-6.640348	2.005517	-0.085205
H	-7.715382	2.144127	0.020122
H	-6.154119	2.619360	0.677972
H	-6.339507	2.383072	-1.072918
B	-1.272374	1.468232	0.033542
C	3.401214	-0.574234	-0.005775
C	4.462845	-1.472817	-0.017489
C	3.639358	0.830023	0.002697
C	5.769507	-0.982997	-0.020968
H	4.296262	-2.544157	-0.023959
C	4.943249	1.305762	-0.000849
C	6.020978	0.397349	-0.012785
H	6.606544	-1.671081	-0.030064
H	5.146221	2.370846	0.005471
B	2.256532	1.553397	0.015121
C	7.367610	0.876519	-0.016606
N	8.456371	1.266229	-0.019717
H	2.051556	2.722736	0.024277
H	-1.117846	2.646494	0.038043

3. Transition Dipole Moments:

System	μ^{00}	μ^{01}	μ^{10}	μ^{11}
B5RO	3.12	1.88	3.31	8.61
B6RO	2.88	0.38	0.65	5.44
B7RO	2.90	1.09	1.93	9.44
B5RC	3.23	2.30	3.96	6.81
B6RC	3.28	2.33	4.03	7.39

B7RC	3.11	1.96	3.42	8.20
B5RC-NH	3.27	2.05	3.47	6.30
B5RC-O	3.28	2.20	3.76	6.50
B5RC-S	3.36	2.15	3.67	7.00
B5RC-BH	3.36	0.68	1.12	5.91
B5RC-BF	3.39	0.82	1.37	6.04
T5RO	3.25	4.64	2.64	10.11
T5RC-AB	3.34	4.81	2.75	9.11
T5RC-BC	3.26	4.80	2.74	9.60
T5RC-ABC	3.28	4.88	2.81	8.90
T5RC-BH-AB	3.41	0.69	1.15	5.75
T5RC-BH-BC	3.44	0.85	1.48	7.89
T5RC-BH-ABC	3.65	0.14	0.23	6.54

Table T1: Different transition dipole moment vectors involved for BP and TP systems.
